# Myocardial Viability: From Proof of Concept to Clinical Practice

**DOI:** 10.1155/2016/1020818

**Published:** 2016-05-29

**Authors:** Aditya Bhat, Gary C. H. Gan, Timothy C. Tan, Chijen Hsu, Alan Robert Denniss

**Affiliations:** ^1^Department of Cardiology, Blacktown Hospital, Blacktown Road, Blacktown NSW 2148, Australia; ^2^Western Sydney University, Richmond, NSW, Australia

## Abstract

Ischaemic left ventricular (LV) dysfunction can arise from myocardial stunning, hibernation, or necrosis. Imaging modalities have become front-line methods in the assessment of viable myocardial tissue, with the aim to stratify patients into optimal treatment pathways. Initial studies, although favorable, lacked sufficient power and sample size to provide conclusive outcomes of viability assessment. Recent trials, including the STICH and HEART studies, have failed to confer prognostic benefits of revascularisation therapy over standard medical management in ischaemic cardiomyopathy. In lieu of these recent findings, assessment of myocardial viability therefore should not be the sole factor for therapy choice. Optimization of medical therapy is paramount, and physicians should feel comfortable in deferring coronary revascularisation in patients with coronary artery disease with reduced LV systolic function. Newer trials are currently underway and will hopefully provide a more complete understanding of the pathos and management of ischaemic cardiomyopathy.

## 1. Introduction

Ischaemic heart disease (IHD) is the leading cause of morbidity and mortality in Western society, with an overrepresentation in the primary healthcare burden [1–3]. In patients with coronary artery disease (CAD), left ventricular (LV) function remains one of the most robust prognostic determinants of survival [4–6], also impacting total hospital separations and many defined quality of life indicators (including physical and social functioning, energy, and general health perception) [7, 8].

The myocardium is exquisitely sensitive to ischemia, with contractile dysfunction occurring shortly after an ischaemic stimulus. The degree of contractile impairment remains strongly under the influence of the severity and duration of the ischaemic event, with irreversible myocardial necrosis representing the end pathway of prolonged and significant coronary ischemia [9]. Hence, the primary priority in the management of acute coronary syndromes is to limit the extent of myocardial necrosis via reperfusion therapies, such as primary angioplasty and thrombolysis, particularly in the setting of electrocardiographic evidence of transmural ischemia.

Despite early intervention, patients with IHD have a predisposition to develop structural heart disease, with impairment of myocardial function leading to cardiac failure, a condition termed as “ischaemic cardiomyopathy” [10]. Given that progressive reductions in LV systolic function secondary to the ischaemic substrate have been shown to be associated with poor outcomes, these aberrations represent a theoretically salvageable pathway via revascularisation.

The ability to distinguish whether dysfunctional myocardium is “viable” and thus able to recover following revascularisation, however, presents a clinical challenge in current practice.

This review examines the concept of myocardial viability, with focus on imaging modalities and principal outcome trials.

## 2. Myocardial Viability: Theoretical Precepts

Viability of myocardial tissue is the central principal which underpins reperfusion therapies, whether in the acute phase following myocardial infarction or in chronic ischemia-mediated LV dysfunction. Should “viable” myocardial tissue be present, restoration of adequate coronary blood flow should in theory improve myocardial performance and LV ejection fraction (EF), with the hope of translating into improved long-term outcomes.

### 2.1. Myocardial Stunning

Early work into CAD and myocardial flow limitation supported the hypothesis that myocardial ischemia results in significant myocyte injury [11].

Heyndrickx and coinvestigators first demonstrated the impact of reversible ischemia on myocardial contractile reserve. Utilising animal models, they demonstrated that short (5- or 15-minute) induced episodes of ischemia to the myocardium, with a subsequent reperfusion period (lasting 6 hours for a 5-minute episode of ischemia, and >24 hours following a 15-minute episode of ischemia), resulted in regional deficits in contractile function that persisted despite reperfusion [12]. This phenomenon, termed as* myocardial stunning*, was defined as a prolonged and completely reversible dysfunction of the ischaemic myocardium that continued after restoration of coronary arterial flow [12]. Stunned myocardium was found to be responsive to inotropes in these early studies, with an increase in contractile function in response to exogenous catecholamines [12].

Myocardial stunning has also been found in clinical practice, particularly in the setting of increased myocardial demand or reduced coronary supply such as following coronary artery spasm, postmyocardial infarction, or postcardiopulmonary bypass secondary to “cardiac off-time.” Myocardial stunning is also prominent in patients following successful revascularisation postinfarct, wherein there is prolonged systolic dysfunction which takes several days to normalise after the incident event [13–15].

### 2.2. Myocardial Hibernation

Myocardial hibernation represents a condition of sustained depression of myocardial function in the setting of CAD, which is amenable to improvement in function postrevascularisation. This term was first coined by Diamond and colleagues in 1978 [11], and was later popularised by the works of Rahimtoola [16]. This sustained depression in myocardial function is hypothesised to be mediated by fundamental changes in myocardial energetics and metabolism, which are both reduced to match a concomitant reduction in coronary flow reserve.

An alternate hypothesis offered for the mechanism of sustained contractile depression is the* repetitive stunning hypothesis*. In this theory, multiple bouts of demand ischemia in context of flow limitation result in repetitive episodes of ischaemic myocardial dysfunction (or stunning), which eventually creates an environment of sustained depression of contractile function [17].

### 2.3. Stunning versus Hibernation

Resting myocardial perfusion is normal or near normal in stunning but is reduced in hibernation. Stunning of the myocardium is frequently represented as transient regional LV wall motion abnormality persisting for hours to days following reperfusion after short-term but significant impairment of coronary blood flow. Hibernating myocardium, on the other hand, is a state of persistently impaired myocardial performance at rest due to a chronic reduction in coronary blood flow that can be restored by favorably altering the supply/demand relationship of the myocardium [18]. Although traditionally described as two separate entities, stunned and hibernating myocardium may in fact represent stages on a continuum of LV dysfunction resulting from repeated ischaemic episodes (as per the repetitive stunning hypothesis).

Identifying myocardial hibernation is of clinical relevance, as it represents potentially salvageable myocardial tissue. Coronary revascularisation in this context is likely to improve contractile performance, LV systolic function, and, in turn, overall morbidity and mortality. However, hibernating myocardium, if left untreated, has the potential to transform into clinically overt heart failure. Revascularisation, via either percutaneous angioplasty or coronary bypass surgery, is the primary avenue of restoring coronary blood flow, unless natural collaterals are formed from the primary diseased vessel.

## 3. Methods of Viability Assessment

### 3.1. Electrocardiography

Pathologic Q waves, deep initial negative deflections of the QRS complex, were traditionally thought to be secondary to chronic transmural ischemia and representative of “dead myocardium.” On subsequent analysis, it has been demonstrated that presence of pathologic Q waves has a poor correlation with the lack of residual viable myocardial tissue, with a relatively low sensitivity (41–65%) and specificity (69–79%) relative to other imaging modalities [19, 20].

Utility of exercise electrocardiography improves viability detection, with elevation of the ST segment during exercise in infarct-related leads being representative of viable myocardium (sensitivity 82% and specificity 100%) [21]. A similar finding is appreciated when evaluating reciprocal ST segment depression associated with exercise-induced ST elevation, with comparable sensitivity and specificity in viability recognition (84% and 100%, resp.) [22].

Use of normalisation of abnormal T waves during exercise electrocardiography for viability assessment, on the other hand, has conflicting reports in the literature [23, 24], with more recent trials showing poorer sensitivities [25, 26].

### 3.2. Echocardiography

#### 3.2.1. Echocardiography: LV Morphology

Assessment of echocardiographic parameters at rest is important in assessment of viability. Severe dilatation of the LV is a marker of nonviable myocardium, with higher end-systolic volume indices associated with poor ventricular functional recovery [27]. These findings portend to a poorer prognosis, with left ventricular end-systolic volumes ≥130 mL having a reduced 3-year survival rate [27]. The thickness of the LV wall has also been shown to be predictive of viability, with a thin LV wall representative of nonviable tissue or scar in patients with CAD [28]. Studies have shown that end-diastolic wall thickness less than 5-6 mm indicates lack of contractile reserve [28], with end-diastolic wall thickness ≥5 mm on two-dimensional echocardiographic measurements having a sensitivity of 100% and specificity of 28% in prediction of improvement in contractile function twelve months following surgical revascularisation in patients with LV impairment (LVEF < 50%) [29]. In keeping with these findings, Cwajg and colleagues (2000) also found that an end-diastolic wall thickness >6 mm was predictive of contractile recovery following revascularisation with a sensitivity of 94% and specificity of 48%, while segments with an end-diastolic thickness of <6 mm rarely have contractile reserve [29].

#### 3.2.2. Echocardiography: Dobutamine Stress Echocardiography

Dobutamine stress echocardiography (DSE) is a valuable tool in the assessment of viability of the myocardium. Classically, four responses are noted in a dysfunctional myocardial response to dobutamine. These are as follows [30–33]:

(i) Biphasic response: low-dose dobutamine (defined as 5–10 *μ*g/kg/min) can increase contractility in dysfunctional segments which are still viable. At higher doses (10–40 *μ*g/kg/min), wall motion in these segments may further improve or paradoxically diminish, reflecting tachycardia-induced ischemia. This phenomenon is referred to as a* biphasic response* and has been shown to be highly predictable of functional recovery postrevascularisation. This finding is suggestive of limited, but present, myocardial reserve in the hibernating myocardium.
(ii) Worsening contractile function with lack of initial improvement with dobutamine: this response is suggestive of a hibernating myocardium which is supplied by a critically limited arterial supply, with no contractile reserve.
(iii) Sustained improvement with increasing dobutamine dose: this response is traditionally seen in the setting of myocardial stunning.
(iv) No response to dobutamine: this response is indicative of lack of functional reserve and, thus, lack of viable myocardial tissue.

Dysfunctional areas with resting end-diastolic wall thickness of less than 6 mm are thought to reflect significant scar. They are not known to show functional improvement with DSE and do not improve postrevascularisation.

DSE has been shown to have a sensitivity and specificity range for prediction of contractile recovery that is modestly high (71–97% and 63–95%, resp.), with the biphasic response having the greatest predictive capability of the four responses [33].

#### 3.2.3. Echocardiography: Myocardial Contrast Echocardiography

Myocardial contrast echocardiography (MCE) utilises acoustically reflective high molecular weight inert gases which form microbubbles and act as a contrast agent. These bubbles remain within the intravascular space and help attenuate the borders of the left ventricle. Tissue capillary blood flow, a determinant of myocardial perfusion, is the byproduct of capillary blood volume and myocardial blood velocity.

Once the microbubbles reach a steady-state concentration, high-burst ultrasonography is used to displace the microbubbles, with subsequent replenishment within myocardial segments over the following cardiac cycles reflective of myocardial blood velocity. Segments are deemed viable if there is homogeneity of contrast intensity, which is in keeping with intact myocardial microvasculature. Nonviable segments, however, lack contrast enhancement and represent necrotic myocardial cells causing obstruction and collapse of the microcirculation [33–35].

MCE has been shown to have a high sensitivity (78–90%), however, low specificity (51–72%), of myocardial contractile recovery postrevascularisation relative to DSE (which on average has a relatively high specificity but lower sensitivity) [36–39]. A combination of the two modalities seems to be optimal in echocardiographic assessment of myocardial viability (sensitivity 96% and specificity 63%) [33].

#### 3.2.4. Echocardiography: Strain Analysis

Myocardial deformation indices, including tissue Doppler imaging (TDI) and strain assessment, are new echocardiographic modalities in the assessment of myocardial function, which allow for a more complete appraisal of myocardial motion and overcome traditional challenges of two-dimensional echocardiography with regard to regional myocardial assessment [40, 41]. Strain is defined as the deformation of an object relative to its original location, with strain rate being reflective of the gradient of the velocities between the two locations. This information can be quantified via TDI or two-dimensional speckle tracking.

Myocardial deformation (strain) and deformation rate (strain rate) provide multidimensional evaluation of myocardial mechanics (longitudinal, radial, and circumferential function) and have the added advantage of being able to detect subtle wall motion abnormalities of regional function that do not decrease global LVEF [42, 43]. This, in part, is reflected by the fact that strain rate imaging is of lower load-dependence and hence provides a better measure of contractility. Additionally, it is not affected by global myocardial displacement and the tethering effect of neighboring wall segments which encumber standard two-dimensional visual assessments.

Both TDI and speckle-tracking echocardiography have been shown to be facilitative in prediction of myocardial viability. This is of relevance given the limitations of subjective assessment of wall thickness as well as operator dependence with traditional two-dimensional stress echocardiographic methods. Bansal and colleagues (2010) revealed that longitudinal and circumferential strain and strain rate measurements at rest and low-dose dobutamine concentrations were predictive of functional recovery postrevascularisation using strain-based imaging. Furthermore, only tissue velocity imaging was found to have incremental value over wall motion analysis [44].

Based on a study by Hoffmann et al. (2002), an increase of peak systolic strain rate greater than or equal to 0.23/s had a sensitivity of 83% and specificity of 84% in discerning viable myocardium as determined by ^18^FDG [45]. Additionally, radial strain >9.5% was associated with a sensitivity of 83.9% and specificity of 81.4%, whereas a change in longitudinal strain >14.6% provided a sensitivity of 86.7% and specificity of 90.2% in detection of viable myocardium using strain imaging with adenosine stress echocardiography in a small trial by Ran and colleagues (2012) [46]. Further work into the field is in progress, with several larger trials underway.

Advantages of echocardiography include ease of procedure and widespread availability as well as its noninvasive qualities. Furthermore, with DSE, there is an ability to monitor functional response to accurate uptitration of inotropic therapy. Limitations of echocardiography include its high operator dependency with resultant inter- and intraobserver variability. Patients with comorbidities such as obesity, chronic obstructive airflow limitation, and thoracic chest wall abnormalities limit the acoustic window and thus impair LV views. Furthermore, with respect to DSE, assessment relies heavily on subjective visual interpretation of wall motion abnormalities.

### 3.3. Single-Photon Emission CT

Single-photon emission CT (SPECT) is a modality which utilises radionuclide-labeled tracer compounds to measure myocardial uptake. Initial acquisition signifies delivery of the tracer throughout the circulation. The images acquired following this (usually 4–24 hours later) reflect myocardial sarcolemmal integrity [47].

Primary tracers include ^99m^Tc-sestamibi, ^99m^Tc-tetrofosmin, and ^201^Thallium. These molecules are lipophilic and permeate through myocardial cellular membranes via passive diffusion or active uptake from Na^+^/K^+^ ATPase systems. Intracellular retention, however, requires intact function of the mitochondrion with preservation of the action potential, and as such serves as a marker of viability. These tracer agents emit high-energy photons, which are captured via gated SPECT, and provide information of global LV function and viability of the myocardium [47, 48].

Viability assessment with SPECT can be performed at rest, following physical exercise or chemical coronary stress. With stress testing, physical exertion or chemical agents (specifically, dipyridamole or adenosine) are used. Imaging is performed immediately following the test, with delayed imaging repeated 3 to 4 hours later, allowing for adequate redistribution of the tracer agent. If warranted, imaging may be repeated at 24 hours after stress (termed as* late distribution imaging*) [49].

Viability is seen with myocardial segments which reveal defective uptake immediately following stress, with subsequent replenishment of uptake at 3 to 4 hours. Critically hypoperfused myocardial segments may still be viable if defective uptake is seen at this delayed time-point, warranting repeat imaging at 24 hours after stress to allow for redistribution of the tracer to significantly hypoperfused myocardial regions. Nonviable myocardium reveals fixed defective uptake throughout a 24-hour imaging cycle [49].

SPECT has been shown to provide a higher sensitivity (64–72%) however lower specificity (45–88%) than modalities based on evaluation of residual contractile recovery [49, 50]. Primary limitations include cost, ionising radiation exposure, low spatial resolution, and attenuation artefacts. These artefacts can be removed via integration of multislice CT and SPECT [50].

### 3.4. Positron Emission Tomography

Positron emission tomography (PET) imaging is based on the shift of myocardial perfusion energetics, whereby chronically underperfused myocardial tissue shifts from utilization of free fatty acids (that require high oxygenation for use) to that of glucose metabolism, which uses a more anaerobic process at the expense of poor energetic efficiency. This translates into uptake of perfusion tracers in myocardial segments which are hypoperfused. Perfusion tracers, including ^13^N-labeled ammonia (^13^NH_3_) and ^18^F-fluorodeoxyglucose (^18^FDG), are utilised in standard practice.

**Figure 1:**
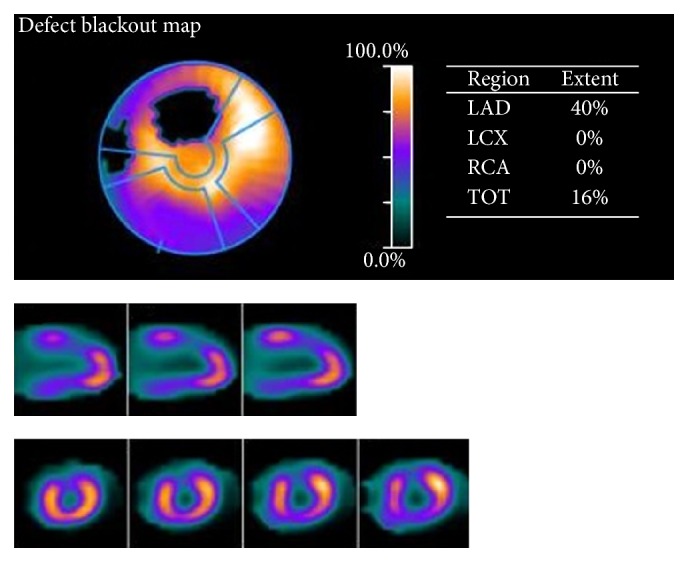
PET assessment. Comment: 59-year-old male with known ischaemic heart disease (requiring bypass grafting) presents for PET assessment in the context of new-onset angina. PET assessment findings of scintigraphic evidence of a reversible perfusion defect of the mid third of the anterior wall is noted. This gated data suggests a high-grade stenosis supplying this region. Noted normal left ventricular systolic function at rest with an inducible wall motion abnormality and significant fall in LVEF with pharmacological stress.

Regions are classified according to the degree of “flow-metabolism” matching, which is reflected by concordance between myocardial blood flow and ^18^FDG uptake. Regions of myocardium where there is a concordance between reduction of myocardial blood flow and ^18^FDG uptake (flow-metabolism match) reflect irreversible myocardial injury. In contrast, areas where FDG uptake (reflective of metabolism) is preserved or increased despite perfusion deficits reflect viable myocardium [51] (Figure 1).

Primary advantages of PET over SPECT include better spatial resolution and superior average sensitivity and specificity (88% and 73%, resp.) [34]. Reduced availability of PET scanners and the variability of FDG uptake are the primary limitations. Many factors, including cardiac output, sympathetic activity, heart failure status, and degree of ischemia, impact FDG uptake and, thus, scan quality [49, 51].

### 3.5. Cardiovascular Magnetic Resonance

Cine cardiovascular magnetic resonance (CMR) sequencing provides information on global left ventricular function and regional wall motion. It can be used in conjunction with dobutamine stress and gadolinium-chelated contrast. Gadolinium-chelated contrast agents have been utilised to detect perfusion deficits, microvascular obstruction, and myocardial scarring. Accumulation of contrast agents have a paramagnetic effect, which form bright signal intensities in areas of accumulation. These agents are unable to penetrate cardiac myocytes with intact membranes; however, they easily diffuse and accumulate into extracellular membranes with increased volume of distribution (e.g., myocardial fibrosis) or ruptured cellular membranes (e.g., acute myocardial infarction) during the “late” steady-state phase [52].

**Figure 2:**
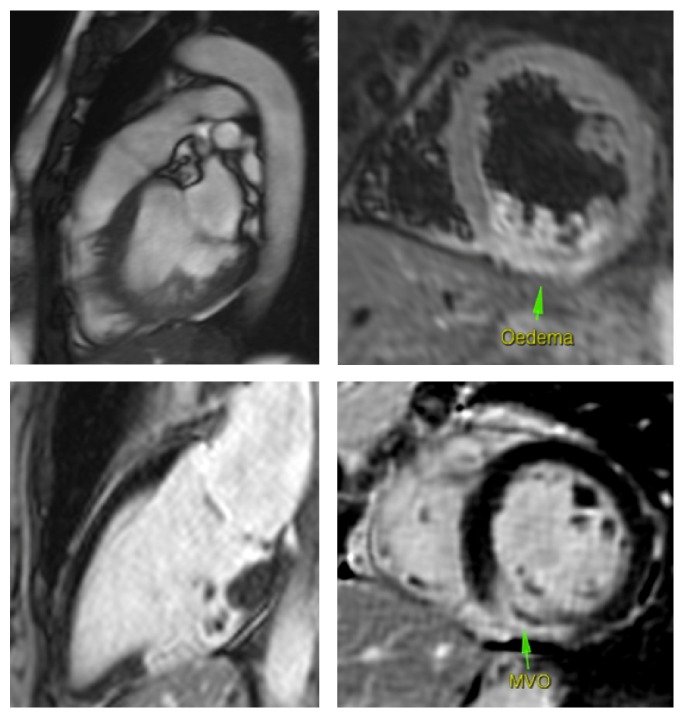
CMR assessment. Comment: 51-year-old female following an inferior ST segment elevation myocardial infarction. CMR revealed hyperintensity in the midinferior wall on T2 weighted images. There is 100% transmural late gadolinium enhancement of the midinferior wall indicating nonviability of this region of myocardium. Of note, an area of hypoenhancement is also present in the middle of the hyperenhancement region, indicating microvascular obstruction. There is also late gadolinium enhancement affecting part of the posterior papillary muscle.

The transmural extent of scarring is inversely correlated with functional recovery of the dysfunctional myocardium postrevascularisation, whereas the absence of late gadolinium enhancement in a hypokinetic myocardium is associated with functional recovery postrevascularisation [52, 53] (Figure 2).

Benefits of CMR over alternate imaging modalities include excellent spatial imaging, ability to discern transmural variations in viability, and provision of accurate quantification of nonviable or necrotic tissue. The ability of CMR for detection of scar (nonviable tissue) is robust, with a sensitivity of 83% and specificity of 88% [49, 54]. Primary limitations of CMR include cost, poor availability, and prolonged study periods requiring patient immobility and breath holding.

A summary of trials evaluating the utility of different imaging modalities in viability assessment is shown in Table 1 [29, 55–64].

## 4. Prognostic Value of Viability Testing

Numerous nonrandomized retrospective studies in the early 1990s evaluated the value of viability testing. A meta-analysis of these trials revealed a significant association between revascularisation and improvement in mortality utilising viability testing in patients with known ischaemic cardiomyopathy. This finding was shared irrespective of imaging modality chosen [55]. Primary limitations of these studies, however, included lack of standardisation and adherence to optimal medical therapy during this period, with outcome reviews having been retrospective in nature. Furthermore, advancement to medical treatment of cardiac failure has improved since these studies, as have techniques of coronary revascularisation.

There was significant clinical uncertainty with regard to the impact of viability on survival given the lack of large, heavily powered randomized trials. These questions were largely addressed in the Surgical Treatment for Ischaemic Heart Failure (STICH) trial (2011). The STICH trial was designed to evaluate the impact of coronary artery bypass grafting (CABG) in management of patients with CAD with reduced LVEF.

## 5. The STICH Trial

In this multicenter (127 clinical sites), nonblinded, randomized trial, 1212 participants were enrolled, with 601 undergoing myocardial viability assessments. Participants were enrolled on the basis of echocardiographic evidence of LV systolic dysfunction (defined as LVEF ≤ 35%) and coronary angiography revealing CAD amenable to surgical intervention. Myocardial viability assessment was provided via DSE (*n* = 130), or SPECT (*n* = 321), or both (*n* = 150). Of the viability subgroup, 298 participants were randomly assigned to receive medical therapy plus surgical revascularisation (cardiac bypass) and 303 received solitary medical management. Participants were followed up at intervals (time of discharge or at 30 days, every 4 months within the first year, and every 6 months thereafter) with a median length of follow-up of 56 months (minimum 12 months, maximum 100 months) [56].

Despite an association of viable myocardium to likelihood of survival in this cohort, multivariate analysis did not find a statistically significant mortality benefit with surgical intervention (*p* = 0.21). Furthermore, assessment of myocardial viability did not provide a differential benefit for surgical intervention (*p* = 0.53). That is to say that viability assessment did not recognize participants who would benefit from CABG relative to medical therapy [56].

Secondary endpoints were more forgiving towards revascularisation, with bypass surgery having a significant reduction in cardiovascular mortality (28% versus 33%; *p* = 0.05), composite death from any cause and hospitalization from cardiovascular causes (58% versus 68%; *p* < 0.001). Long-term follow-up (>4 years) of both cohorts revealed a reduction in all-cause mortality in the surgical revascularisation cohort compared to solitary medical therapy; however, this finding was not statistically significant (*p* = 0.12). These positive secondary findings should be interpreted with caution given a negative primary outcome measure [56].

This trial was not, however, without its limitations. Firstly, randomization was not performed on the basis of viability which represented a potential selection bias. Secondly, there was a differential effect on participant profile and viability, with a high proportion of participants (81%) in the viability subgroup having single-vessel disease. Given the scope of the paper (medical therapy versus surgical intervention), this differential profile may have selected out participants for whom viability assessment may not have been required. Thirdly, analysis in this study was limited to DSE and SPECT modalities, with no analysis of PET or CMR on viability assessment. This creates difficulty with extrapolation of these results to other imaging modalities of viability assessment.

Despite these limitations, this study represents the largest analysis of the influence of myocardial viability on clinical endpoints in persons with ischaemic cardiomyopathy to date,and was the first to assess the differential effect of viability on revascularisation versus medical management.

## 6. The HEART Trial

The Heart Failure Revascularisation Trial (HEART) (2011) was a multicenter study comparing the efficacy of surgical revascularisation with optimal medical treatment in the management of persons with clinically diagnosed cardiac failure with reduced EF (LVEF < 35%) and evidence of CAD. Participants were screened for viable myocardium via DSE. An inclusionary prerequisite was the presence of at least 5 viable LV segments with reduced contractility using a 17-segment model [57].

138 participants were randomized to interventional (*n* = 65) and medical arms (*n* = 69) and followed up over a five-year period. The primary outcome revealed noninferiority of medical therapy. This study was, however, underpowered secondary to a relatively small sample size. Furthermore, the primary modality of viability assessment was DSE, which has a lower sensitivity for viability detection relative to other imaging modalities. Additionally, randomization had not occurred prior to viability assessment, therefore clouding the impact of viability assessment on treatment outcomes [57].

## 7. PARR-2 Trial

The PET and Recovery Following Revascularisation-2 (PARR-2) trial (2007) evaluated the efficacy of perfusion FDG-mediated PET imaging in risk stratification and identification of patients who would most benefit from revascularisation [58].

The study enrolled 430 participants, with an inclusionary criterion of a LVEF <35% and suspected or confirmed CAD. Participants were randomly placed to receive FDG and perfusion PET imaging versus standard care (i.e., no FDG imaging). Effect of PET scanning on appropriate decision showed a nonsignificant trend towards a reduction in the predefined composite endpoint (cardiac death, myocardial infarction, or cardiac rehospitalization) at one year (Hazard Ratio 0.78, 95% CI 0.58 to 1.1; *p* = 0.15), with post hoc analysis showing a statistically significant reduction in adverse events in the FDG PET-assisted group (Hazard Ratio 0.62, 95% CI 0.42 to 0.93; *p* = 0.019) [58].

The key limitation of the study involved poor adherence to therapeutic strategy, with only 75% of participants treated accordingly to viability imaging.

## 8. Ottawa-FIVE Substudy

The Ottawa-FIVE substudy (of the PARR-2 trial) (2010) evaluated 111 participants with LV systolic dysfunction (specifically, persons with LVEF < 35%) and suspected or confirmed CAD in a single center with experience with FDG PET imaging [59]. A statistically significant reduction in the primary composite endpoint (cardiac death, myocardial infarction, or cardiac rehospitalization) was found within the FDG PET-guided therapy group in comparison with the standard-therapy arm (19% versus 41%, Hazard Ratio 0.34, and 95% CI 0.16 to 0.72; *p* = 0.005). The results of this substudy illustrated prognostic benefit with the utilization of FDG PET viability imaging in ischaemic cardiomyopathy when used in centers with experience in PET imaging [59].

Despite the relatively disappointing results of the aforementioned trials, the 2013 American Heart Association/American College of Cardiology guidelines for management of heart failure remain unaltered in their Class IIa (Level of Evidence B) recommendation for viability testing in the work-up for revascularisation in patients with ischaemic cardiomyopathy. This is in keeping with the belief that there may still be diagnostic and prognostic benefit in the utility of viability studies which have not become apparent given the limitations of the aforementioned primary trials.

## 9. Conclusion

Ischaemic LV dysfunction can arise from myocardial stunning, hibernation, or necrosis. In line with technological advances, noninvasive imaging modalities have become front-line methods in the assessment of viable myocardial tissue, with each modality conferring a variable advantage in terms of sensitivity and specificity, culminating in the overriding goal of accurate stratification of patients into optimal treatment pathways.

Despite determined research efforts, however, many questions remain unanswered with regard to myocardial viability. Initial studies, although favorable, lacked sufficient power and sample size to provide conclusive outcomes of viability assessment. More recent trials, including the STICH and HEART studies, have failed to confer prognostic benefits of revascularisation therapy over standard medical management in ischaemic cardiomyopathy but have their own limitations. In lieu of these recent findings, however, assessment of myocardial viability therefore should not be the arbitrating factor for therapy choice. Optimization of medical therapy for all patients is paramount, and physicians should feel comfortable in deferring coronary revascularisation in patients with CAD with reduced LVEF at present.

It is clear that further trials are needed to better our understanding of the mechanistic underpinnings of the viable myocardium as well as the underlying pathos of ischaemic cardiomyopathy. Newer trials such as the AIMI-HF (Alternative Imaging Modalities in Ischaemic Heart Failure) study, the largest randomized trial to date evaluating the role of imaging in the treatment of ischaemic cardiomyopathy, are currently underway and will hopefully decipher some of these uncertainties [65].

## Figures and Tables

**Table 1:** Summary of studies evaluating improvement in segmental myocardial function with revascularisation.

Study	Period	Study design	Setting (center)	Patient (*n*)	Modality of viability assessment	Sensitivity	Specificity
Arnese et al. [55]	1995	Prospective	Single	38	Stress TTE, PET	74, 89	95, 48
Cornel et al. [56]	1998	Prospective	Multi	61	Stress TTE	89	81
Pagano et al. [57]	1998	Prospective	Single	30	Stress TTE, PET	60, 99	33, 62
Bax et al. [58]	1999	Prospective	Single	68	Stress TTE	89	74
Pasquet et al. [59]	1999	Prospective	Single	94	Stress TTE, PET	69, 84	78, 37
Baer et al. [60]	2000	Prospective	Single	103	CMR, Stress TOE	86, 82	92, 83
Wiggers et al. [61]	2000	Prospective	Single	46	PET, Stress TTE	81, 51	56, 89
Cwajg et al. [29]	2000	Prospective	Single	45	PET, Stress TTE	91, 94	50, 48
Schmidt et al. [62]	2004	Prospective	Single	40	CMR, PET	96, 100	87, 73
Hanekom et al. [63]	2005	Prospective	Single	55	SRI, TTE	78, 73	77, 77
Slart et al. [64]	2006	Prospective	Single	47	DISA SPECT, PET	89, 90	86, 86

TTE, transthoracic echocardiography; TOE, transesophageal echocardiography; PET, photon emission tomography; CMR, cardiac magnetic resonance imaging; SRI, strain rate imaging echocardiography; DISA SPECT, dual-isotope simultaneous acquisition (DISA) SPECT.
